# Cattle aggregations at shared resources create potential parasite exposure hotspots for wildlife

**DOI:** 10.1098/rspb.2023.2239

**Published:** 2023-12-06

**Authors:** Georgia Titcomb, Jenna Hulke, John Naisikie Mantas, Benard Gituku, Hillary Young

**Affiliations:** ^1^ Department of Fish, Wildlife, and Conservation Biology, Colorado State University, Fort Collins 80523-1019, CO, USA; ^2^ Department of Ecology, Evolution, and Marine Biology, University of California, Santa Barbara, CA, USA; ^3^ Department of Biology, Texas A&M University, College Station, TX 77843, USA; ^4^ Mpala Research Centre, Laikipia County, Nanyuki, Kenya; ^5^ Ecological Monitoring Unit, Ol Pejeta Conservancy, Nanyuki, Kenya

**Keywords:** gastrointestinal parasite, watering hole, multi-pathogen sharing, helminth, livestock–wildlife interface

## Abstract

Globally rising livestock populations and declining wildlife numbers are likely to dramatically change disease risk for wildlife and livestock, especially at resources where they congregate. However, limited understanding of interspecific transmission dynamics at these hotspots hinders disease prediction or mitigation. In this study, we combined gastrointestinal nematode density and host foraging activity measurements from our prior work in an East African tropical savannah system with three estimates of parasite sharing capacity to investigate how interspecific exposures alter the relative riskiness of an important resource – water – among cattle and five dominant herbivore species. We found that due to their high parasite output, water dependence and parasite sharing capacity, cattle greatly increased potential parasite exposures at water sources for wild ruminants. When untreated for parasites, cattle accounted for over two-thirds of total potential exposures around water for wild ruminants, driving 2–23-fold increases in relative exposure levels at water sources. Simulated changes in wildlife and cattle ratios showed that water sources become increasingly important hotspots of interspecific transmission for wild ruminants when relative abundance of cattle parasites increases. These results emphasize that livestock have significant potential to alter the level and distribution of parasite exposures across the landscape for wild ruminants.

## 1. Introduction

Cattle now account for 35% of mammal biomass on the planet [1] and their populations have grown by 60% over the past six decades [2]. At the same time, wildlife numbers have dropped dramatically [3,4] such that wild mammals account for a mere 4% of mammal biomass [1]. Substantial research has investigated ways to promote wildlife and cattle coexistence to conserve wildlife while still providing economically beneficial outcomes for people, often by minimizing competition or encouraging facilitation across food resources [5,6]. Considering that the majority of pathogens and parasites have multiple hosts [7], it is especially important to account for potential parasite sharing between wildlife and cattle at hotspots that attract and aggregate many different animals [8,9]. In arid locations in particular, water sources can be significant foci of faecal-oral parasite transmission [10], but the extent to which they foster cross-species transmission is largely unexplored.

Multi-host pathogen sharing is particularly important at resources that draw together many different species at the livestock-wildlife interface [11]. Water sources in particular can concentrate an array of different host species that then can be exposed to high parasite levels via drinking or foraging for food nearby [9,12]. Akin to superspreading individuals, such environmental transmission hotspots are important because overall transmission dynamics can be heavily influenced by a small fraction of the landscape [8,13]. In addition, similar to identifying and focusing management on superspreading individuals for directly transmitted diseases, hotspot identification and management can improve efficiency in addressing environmentally transmitted diseases [8]. However, quantifying heterogeneity in transmission risk across landscape features is particularly challenging for many environmentally transmitted parasites, as it involves integrating data on parasite density across large spaces with fine-scale measurement of host contact rates over time. In the case of multi-host parasites (the majority of all parasites [7]), information about the susceptibility of different host species is also required. Thus, we have very little empirical data on multi-host dynamics at hotspots of environmentally transmitted parasites.

Wildlife and livestock host numerous pathogens that may substantially impact one another, including viruses, bacteria, ectoparasites and many gastrointestinal parasites. Wildlife, and particularly wild ungulates, are important reservoir hosts for several diseases of cattle, such as brucellosis, babesiosis, and foot and mouth disease [14,15]. Likewise, livestock may harbour parasites and pathogens that influence wildlife health. For example, while cattle are primary hosts of bovine tuberculosis, the bacterium has also spread to and circulates among a wide range of other species [16]. Conversely, livestock health management can help reduce disease risk for wildlife; for example, acaricide use to reduce tick burdens has been shown to lower tick populations across a landscape [17]. For environmentally transmitted parasites like gastrointestinal helminths, cross-species transmission is heightened between closely related and sympatric species [10,18,19]. Many environmentally transmitted parasites exhibit density-dependent transmission [20], in which higher host density leads to increased transmission. Thus, overlapping host species, especially closely related species, can increase the density of suitable hosts, potentially driving higher infection rates. For example, one study found that in areas where multiple wild bovid species overlapped, strongyle nematode abundance and richness were elevated in areas where their habitats overlapped [21]. Therefore, failure to consider parasite sharing among multiple host species can lead to underestimates of parasite transmission [22].

The potential for parasite sharing at water sources is relevant globally given that drylands account for 41% of the Earth's land surface [23], and East African tropical savannahs are an especially important context for investigating interspecific parasite transmission among multiple herbivore species. Many wildlife species of conservation concern overlap with larger livestock ranching operations or smaller scale community grazing [6] and are often supported by provisional water sources that concentrate water-dependent animals [10]. In keeping with global patterns [1], this area is also experiencing consistent biomass shifts in favour of livestock. For example, aerial wildlife counts have shown that livestock biomass in Kenya was 8.1 times that of wildlife in 2011–2013 compared to 3.5 times the large wild herbivore biomass in 1977–1980 [4].

Wild herbivores are infected by a diverse array of faecal-orally transmitted parasites that have highly variable effects on host health, some of which are also globally important parasites of livestock (e.g. *Haemonchus contortus, Trichostrongylus axei, Cooperia oncophora,* among many others) [10,24]. While diverse parasite communities are important components of biodiversity [25,26] several gastrointestinal nematodes cause substantial morbidity in domestic herbivores and drive large economic losses on a global scale [27]. Most gastrointestinal nematodes are spread when adult worms release thousands of eggs into the landscape upon host defaecation. Parasite eggs develop into infectious larval stages in the environment before infecting herbivorous mammals via drinking or grazing grass that larvae have ascended (e.g. strongylid nematodes [28]). Notably, several dominant gastrointestinal parasites of wildlife are shared with closely related domestic animals or with humans [24,29], and rising rates of anthelmintic resistance raise concerns about future management costs of these parasites in livestock [30].

In this study, we integrated estimates of parasite sharing with our prior work documenting domestic and wild herbivore behaviour and parasite density in this system [9] to characterize multi-host and multi-parasite dynamics at transmission hotspots (water sources) and surrounding non-water ‘matrix’ sites. We compared results using three parasite sharing estimates that differed in their accessibility to researchers in different contexts, exploring similarities in DNA metabarcoding-based methods, host species phylogenetic distance and records in the literature. We quantified and compared intra- and interspecific potential parasite exposures around water sources and matrix sites to test the role of interspecific sharing in creating hotspots of parasite exposure across a landscape. Specifically, we tested our hypothesis that cattle, which are abundant and herded for frequent water access, would account for a substantial share of parasite exposures for closely related wild bovids, and that their high water dependence would amplify water as a parasite exposure hotspot for other species.

## 2. Methods

### (a) Study site

Fieldwork was performed at Ol Pejeta Conservancy (0.0043° S, 36.9637° E), where we surveyed five paired water pans (diameter = 9–10 m and depth = 0.5 m) and ‘matrix’ sites. Surveys were conducted within 150 m of the water pan or centre of the matrix site (total area approx. 7 hectares (ha)), and analyses focused on the inner 50 m where herbivores aggregated (approx. 0.8 ha). We repeated surveys at approximately 3-month intervals from August 2016 to August 2018 (described in [9]). We chose matrix site coordinates by selecting a location 1 km away at a random heading from the focal water pan. The set of possible headings excluded those that would have resulted in a matrix site falling on an airstrip, major road or within 1 km of any other water source. Ol Pejeta Conservancy is home to a robust population of Boran cattle (*Bos indicus × Bos taurus*) and at least 25 large herbivore species, many of which are threatened. We focused on cattle and five dominant wildlife species at Ol Pejeta which together account for 89% of large herbivore camera triggers and 95% of large herbivore dung cover determined from previous work [9]. All five wild herbivores – plains zebra (*Equus quagga*), reticulated giraffe (*Giraffa camelopardalis*), African elephant (*Loxodonta africana*), Cape buffalo (*Syncerus caffer*) and impala (*Aepyceros melampus*) – are either threatened or experiencing population declines [31], and all are often infected by a diverse array of gastrointestinal parasites [29].

### (b) Estimating potential exposures

To test our hypothesis that cattle could account for a substantial share of parasite exposures for related wildlife around water sources, we estimated the degree to which total potential exposures were elevated at water pans compared with matrix sites (a ‘hotspot effect’) when only a single host species was considered (E_intraspecific_) and when intra- and interspecific transmissions were considered (E_total_). Exposure to environmentally transmitted parasites can be estimated as the product of parasite density in the environment, host density and the *per capita* rate of contact (plant consumption in this case) [32]. We chose to focus on exposures using only grazing activity to standardize activity between water sources and matrix sites, as parasite survival likely differs in water versus soil and foliage. However, given that there are likely to be additional exposures from drinking water contaminated with dung, we also consider total exposures under the assumption that drinking activity poses a risk of parasite exposure (electronic supplementary material, appendix SI, §3).

For each focal herbivore species (i) at each of the five water and matrix sites (j), we estimated potential intraspecific exposures *E* (per unit time and area) as the product of parasite density (W) and the density of host grazing (or grazing plus drinking, electronic supplementary material, appendix SI, §3) activity (H), which encompasses both host density and *per capita* contact rate:

2.1 $$E_{{\mathrm{intraspecific}}_{ij}}=H_{ij}\times W_{ij}$$

Where *W_ij_* is the density of parasites (eggs per m^2^) for each focal host species (*i*) and site (*j*) and *H_ij_* is the average aggregate time spent grazing (daily herbivory-seconds per m^2^) for each focal host species and site during a lagged 90-day period following parasite density measurement. We chose a 90-day period to account for variation in herbivore activity between surveys (surveys occurred at approximately 3-month intervals), and because parasites can remain viable in the environment for multiple months [33]. This resulted in 8 sampling events for each of the 5 sites and 2 site types (*n* = 80 total). Of these sampling events, camera traps collected sufficient data for analysis for all but 8 (6 in matrix sites and 2 at water sources).

To account for parasite sharing between each focal host species (i) and each additional host species (*k*), we modified equation 1 by increasing parasite density by the number of parasites contributed by each additional species, weighted by sharing probability.

2.2 $$E_{{\mathrm{interspecific}}_{ijk}}=(W_{ij}+W_{kj}\times \Lambda_{ik})\times H_{ij}$$

Where Λ is a parasite sharing matrix describing the estimated parasite sharing potential between each exposed host species (*i*) and each exposing host species (*k*). To explore the variation in the effect of parasite sharing, we used three different estimates of parasite sharing Λ, described in further detail below: 1) a Bray-Curtis dissimilarity matrix built from parasite DNA metabarcoding data from herbivores at nearby Mpala Research Centre [10], 2) a Jaccard similarity matrix built from host–parasite relationships described in the literature and 3) a phylogenetic distance matrix.

Finally, to compute the total number of exposures, we took the sum of all intra- and interspecific exposures:

2.3 $$E_{{\mathrm{total}}_{ij}}=\sum_{k=1}^{n}(W_{ij}+W_{kj}\times \Lambda_{ik})\times H_{ij}+E_{{\mathrm{intraspecific}}_{ij}}$$

In using these equations to compare relative exposures across sites and species, we made the following assumptions: 1) daily time spent grazing was proportional to consumption rate, 2) parasite development and survival were consistent across sites, and 3) the same proportion of susceptible individuals was found at water and matrix sites.

### (c) Data for parameters

#### (i) Parasite density (*W*)

**Figure 1.**
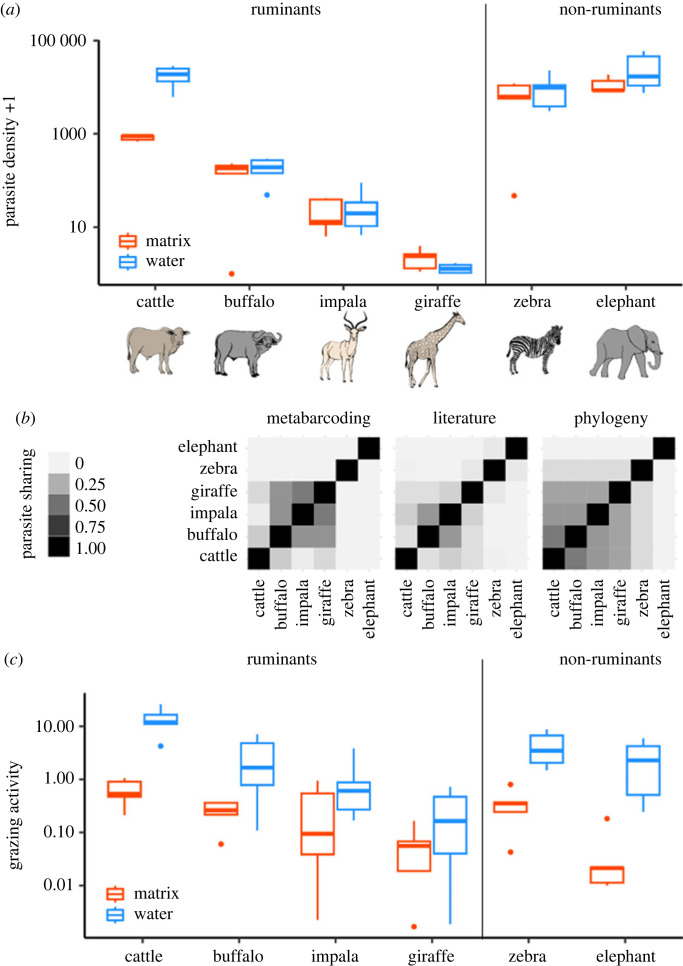
(*a*) Parasite density (eggs per m^2^) for each focal species at permanently filled water pans and matrix sites. (*b*) Three parasite sharing matrices considered in this study, with higher/darker values indicating higher probability of parasite sharing: Bray-Curtis dissimilarities (subtracted from 1) based on local DNA metabarcoding data [10], Jaccard similarities based on parasite presence/absence data reported in the literature [34,35], and scaled phylogenetic distances (subtracted from 1) [36]. (*c*) Herbivore grazing activity (daily individual-seconds per m^2^) for each focal species at permanently filled water pans and matrix sites. Boxplots show values for each site (*n* = 5), averaged over sampling period (*n* = 9) and are displayed on a log_10_ scale for visibility. Panels *a* and *c* are drawn using data published in [9].

To estimate parasite density (eggs per m^2^) (figure 1*a*) we combined data from dung surveys and faecal egg floats. These methods are described in detail in [9] and provided in electronic supplementary material, appendix SI. In brief, we combined dung density measurements from field surveys within 50 m of water or the centre of the matrix site with parasite density measurements. Cattle at Ol Pejeta Conservancy are regularly treated with anthelmintics (parasite egg prevalence = 0.29 [19]); therefore, to estimate the effect of less comprehensively treated cattle on parasite sharing opportunities, we used parasite infection data from nearby Mpala Research Centre, where weaning cattle are dewormed and adults are treated sporadically thereafter.

#### (ii) Parasite sharing (Λ)

We considered three different methods of calculating parasite sharing to examine the robustness of results to variation in sharing estimates (figure 1*b*). A secondary goal of this analysis was to also identify methods that may be more practical in resource-limited contexts. First, we used a Bray-Curtis dissimilarity matrix calculated from a DNA metabarcoding network of strongylid parasites in herbivore dung collected from Mpala Research Center, less than 40 km away [10]. Specifically, the distance matrix was calculated using the pairwise dissimilarity of mean parasite read abundances per host species. Because metabarcoding cannot differentiate between infective parasites and low levels of parasites that are consumed and passed by hosts without infecting them, we excluded mean values less than 2%, as described in [10]. We also excluded one case of an exclusively equid parasite, *Cylicostephanus minutus,* that was found at 2.1% relative read abundance in giraffes. We then converted dissimilarities to a similarity matrix by subtracting the scaled distance matrix from 1. Second, we retrieved host-parasite records from the London Natural History Museum's database [34] for the focal species in our study using the *helminthR* package [35]. We restricted the search to faecal-orally transmitted nematode parasites identified to species. We then computed Jaccard similarities for each pairwise combination using the *vegan* package [37]. Third, we pruned a mammal phylogenetic tree [36] to species in our study and computed all pairwise phylogenetic distances. We then scaled this distance matrix from 0 to 1, with the maximum distance (elephant – giraffe) set to 1. Finally, to give more closely related species a higher probability of parasite sharing than more distantly related species, we subtracted the scaled distance matrix from 1.

#### (iii) Density of host grazing activity (*H*)

We measured herbivore activity and density using camera traps deployed at each water pan and matrix site (*n* = 10; detailed in [9] and summarized in electronic supplementary material, appendix SI). In brief, we set one camera (Moultrie A30, 50° field of view) within 50 m of each water source and matrix site centre for a 2-year period from August 2016–August 2018, servicing cameras monthly (trap nights for these two site types = 3834). We ensured that across sites, cameras had similar detection distances (between 12 m and 15 m) using walk tests. With the assistance of volunteers from the Zooniverse online citizen science platform, we counted animals that were present, grazing and drinking in photographs. Bursts of images that fell within a 5-minute period were grouped into a single trigger. We determined daily grazing activity per m^2^ by integrating activity over trigger sequences and summing all grazing activity within a day. While data validation showed that species identities were highly accurate (91–99%, [9]), we found that the accuracy of grazing activity varied by species, with 90% and 16.5% of instances of giraffe and elephant ‘grazing’ showing browsing, drinking or walking instead (100% of impala grazing instances were accurate). Therefore, giraffe and elephant grazing activity was reduced by 90% and 16.5%, respectively.

While it has been shown that trichostrongylid nematodes migrate up and down vegetation and soil in response to temperature fluctuations [38,39], potentially avoiding hosts when they graze in unfavourably hot conditions, we considered grazing activity at all periods of the day to contribute equally to risk of exposure. We chose to do so because we lack data on the specific temperatures at which larvae move in response to external conditions in this system, and given that prior research has found that moisture, light and parasite species are also important factors [39]. However, we do investigate potential differences in grazing behaviours as a function of temperature and season in electronic supplementary material, appendix SI (electronic supplementary material, table S1, figure S1 and figure S2), finding that grazing activity is relatively consistent across seasons, and that while grazing activity tends to be higher during warm periods in the middle of the day, this pattern does not significantly differ at matrix sites and water, thus leading to similar conclusions about the potential for water sources to act as transmission hotspots relative to matrix sites.

### (d) Analyses

To compare all potential intra- and interspecific exposures across all species at water sources and matrix sites, we fit generalized linear mixed-effects models (GLMMs) to exposure data from equation 1 and equation 2 for each focal host species, using exposing host, site type, and their interaction as fixed effects and site (*n* = 5) and period (*n* = 9) as random effects using the *glmmTMB* package [40]. We repeated this analysis for each of the three methods to calculate parasite sharing.

To compare intraspecific and total exposures for each focal species at water, we fit a single GLMM to exposure data from equation 1 and equation 3 with focal host species, exposure calculation method (intraspecific only or total), site type (matrix or water), and their interaction as fixed effects and site (*n* = 5) and period (*n* = 9) as random effects. We repeated this analysis for each sharing method.

Exposure data for both sets of analyses were highly right-skewed, continuous and contained numerous zeroes, so we used a Tweedie error structure in all GLMMs. We then generated back-transformed estimated marginal means and 95% confidence intervals using the *emmeans* package [41]. We performed *post hoc* tests to determine whether each interspecific exposure differed significantly from intraspecific exposures for each site type, adjusting for multiple comparisons using a Holm correction (*n* = 3 comparisons per focal species and site type).

To estimate the degree to which water sources served as parasite exposure hotspots across a landscape, we followed methods outlined in [9]. We weighted exposure ratios at water versus matrix sites by the proportion of land falling within 50 m of water sources versus the remaining study area (determined using ArcGIS Pro). In doing so, we assumed that the effect of water in strongly aggregating exposures had attenuated after 50 m. We also explored this effect using dung density averages within 150 m of water and under the assumption that grazing activity at 150 m was similar to grazing activity at 50 m.

To explore how changes in the density of cattle parasites in the environment (achieved either through changes in cattle density and/or anthelmintic treatment practices) influenced this pattern, we re-ran analyses with uniform changes in cattle parasites across the landscape in 10% increments. Additionally, to place results in the context of recent regional and global wild mammal declines, we then uniformly reduced wildlife activity and dung density to achieve livestock-to-wildlife biomass ratios that were equivalent to the 1977–1980 and 2011–2013 measurements reported in [4]. Wildlife biomass at Ol Pejeta Conservancy is approximately 1.2 times higher than cattle biomass (calculated from Ol Pejeta aerial survey counts and PanTHERIA biomass estimates [42]); therefore, we reduced wildlife activity and dung density to 22% of baseline values for the 3.5 ratio, and 9.7% of baseline values for the 8.1 ratio.

Finally, we visualized the proportion of total potential exposures contributed by each host species to each focal host species at water sources versus matrix sites by constructing a directed network for each site type (averaging over period and site) and assigning the proportion of total exposures from model estimates as edge weights using *igraph* [43].

Analyses were performed in R v. 4.2.1 [44].

## 3. Results

**Figure 2.**
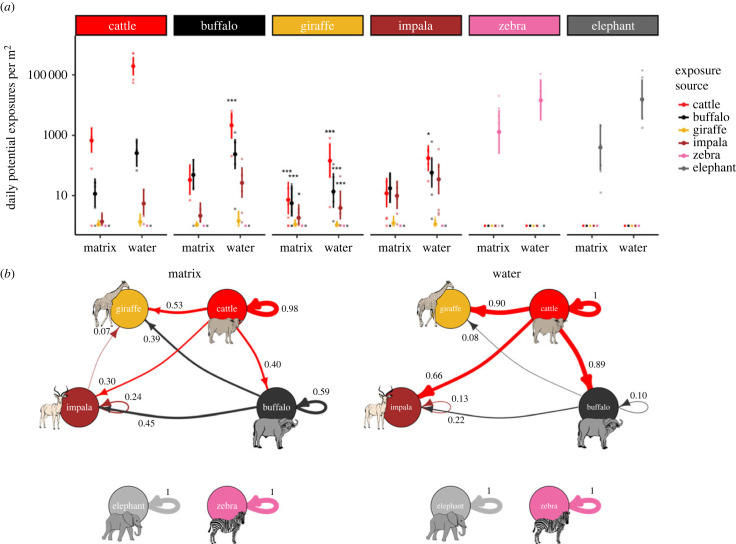
(*a*) Total daily potential parasite exposures for each focal species (panels) and exposing host (coloured points and error bars) at water sources and matrix sites using parasite sharing estimates from metabarcoding data. Points represent site averages, while error bars show means and 95% CIs from model estimates. Colours within each focal host panel show the contribution of each host species to exposures for the focal host, with colour mapping indicated by the panel headings. Interspecific exposures that were significantly higher than within-species exposures are denoted by asterisks (*p* < 0.001, *p* < 0.01 and *p* < 0.05, electronic supplementary material table S3). Data are shown on the log_10_ scale to capture the large variation in potential exposures across species which is driven by variation in population size and parasite output. (*b*) Proportion of total potential exposures contributed by each exposing host at matrix sites and water sources using parasite sharing estimates from metabarcoding data. Arrows representing less than 5% of a species' total exposures are excluded for readability. Plots using parasite sharing data from literature and phylogeny are provided in electronic supplementary material figure S3.

The largest share of potential parasite exposures for wild ruminants in our study (impala, buffalo and giraffe) appeared to come from other species, and this was particularly true around water sources, where cattle drove significantly elevated levels of parasite exposure around these resources (figure 2, electronic supplementary material, table S2, table S3).

### (a) Intra- versus interspecific exposures

Buffalo had similar levels of potential exposure to parasites from cattle as parasites from other buffalo at matrix sites (buffalo/cattle ratio = 1.49, 3.05 and 0.42; *p* = 0.53, 0.09 and 0.26 for contrasts of estimated marginal means from GLMMs using metabarcoding, literature and phylogeny sharing methods respectively; figure 2, electronic supplementary material, table S2, table S3), but this dramatically differed at water sources, where potential parasite exposures were approximately six times higher for parasites from cattle than from buffalo (buffalo/cattle = 0.11, 0.23 and 0.03, *p* < 0.001, *p* = 0.005 and *p* < 0.001 for metabarcoding, literature and phylogeny GLMM contrasts). For impala, potential exposures from cattle, buffalo and other impala were relatively similar at matrix sites, but exposures from cattle dominated at water sources (impala/cattle = 0.20, 0.05 and 0.01; *p* = 0.006, *p* < 0.001, *p* < 0.001 for metabarcoding, literature and phylogeny GLMM contrasts; figure 2, electronic supplementary material, table S2, table S3). Meanwhile, intraspecific exposures for giraffe were consistently lower than interspecific exposures from all three other ruminants across site types, with cattle dominating potential exposures at water sources (giraffe/cattle < 0.001; *p* < 0.001 for GLMM contrasts of all three sharing methods). Together, cattle accounted for approximately 40%, 30% and 53% of potential exposures at matrix sites for buffalo, impala and giraffe, while they accounted for 89%, 66% and 90% of potential exposures at water sources for these three animals (figure 2*b*).

Using metabarcoding data, we estimated that parasites from zebras and elephants did not contribute to total exposures for ruminants. However, while broad patterns were highly consistent across sharing methods (electronic supplementary material, table S2, table S3), literature and phylogeny distance matrices contained very small but non-zero sharing probabilities among elephants, zebra and ruminants, which, when coupled with the high parasite faecal egg counts and dung density in the environment, resulted in increased overall exposures and interspecific sharing relative to values calculated using metabarcoding data (electronic supplementary material, figure S3).

Intra- and interspecific parasite exposure comparisons were similar when we considered drinking water as an additional transmission route (electronic supplementary material, appendix SI, §3, table S5, figure S4).

### (b) Effect of interspecific sharing on parasite exposure hotspots

**Figure 3.**
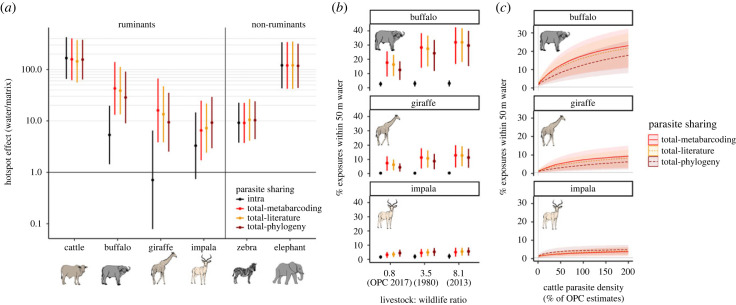
(*a*) Ratios ± 95% CIs of potential parasite exposures at water sources versus matrix sites using different estimates of parasite sharing. The horizontal line at 1 indicates that exposures are equal at water and matrix sites. Black error bars denote estimates without any consideration of interspecific parasite sharing. Cattle, zebra and elephant all had substantially more potential exposures near water than matrix sites, and this was consistent across all methods of calculating parasite sharing. However, wild ruminants experienced greater parasite exposure levels around water sources after accounting for interspecific sharing. (*b*) Simulated reduced wildlife activity and dung density to achieve large-scale livestock: wildlife biomass ratios caused increases in relative levels of parasite exposure at water sources compared with matrix sites for wild ruminants, particularly for buffalo. This was driven by declines in potential exposures at matrix sites and negligible changes to potential exposures around water sources. (*c*) Simulated effects of increasing or decreasing cattle parasite density in the environment on the percentage of parasite exposures at water sources for wild ruminants. The 100% reference refers to field-measured data on cattle dung density at Ol Pejeta Conservancy (OPC) assuming minimal anthelmintic treatment (median eggs per gram = 255).

Our contrasts of total exposures versus intraspecific exposures at water sources and matrix sites showed dramatically increased levels of parasite exposure at water for buffalo, giraffe and impala after considering sharing, while cattle, elephants and zebra were relatively unaffected by interspecific parasite sharing (figures 2 and 3*a*, electronic supplementary material, table S2 and table S3). For buffalo, water sources accounted for 5.3 times as many intraspecific exposures per unit area compared with matrix sites; after accounting for interspecific sharing, this figure rose to 43 times as many total exposures around water compared with matrix sites (see electronic supplementary material, table S4 for all GLMM contrasts). For impala, 3.3 times as many intraspecific exposures at water versus matrix sites rose to 6.5 times as many total exposures; and for giraffe, fewer (0.7 times as many) intraspecific exposures at water climbed to 16 times more total exposures (figure 3*a*).

After accounting for the proportion of the landscape that fell within 50 m of non-riparian water sources (just 0.5% at Ol Pejeta Conservancy), and under the assumption that matrix site measurements represented remaining areas of the landscape, we estimated that approximately 18%, 7% and 3% of all potential parasite exposures occurred within 50 m of water for buffalo, giraffe and impala, respectively; an increase from 2.5%, 0.4% and 1.6% for all of these animals when considering intraspecific exposures alone (electronic supplementary material, table S4). For cattle, elephants and zebras, this percentage was not significantly altered by interspecific exposures, but remained above 35% for elephants and cattle and 4.4% for zebra. Assuming similar patterns of grazing activity up to 150 m from water (2.6% of land cover), the percentage of total exposures that occurred at water reached 32%, 18% and 9% for buffalo, giraffe and impala, respectively, while this percentage was greater than 30% for elephants, cattle and zebra (electronic supplementary material, table S4), in keeping with estimates of intraspecific exposures alone [9]. Removing and increasing cattle parasites had strong effects on this pattern, particularly for buffalo, indicating the significant potential for cattle to influence buffalo parasite dynamics and landscape of parasite risk (figure 3*c*).

Finally, we found that by simulating reductions in wildlife activity and dung density to mirror wildlife and livestock biomass ratios for the broader region, the percentage of potential parasite exposures around water increased (figure 3*b*). Specifically, we found that by reducing wildlife activity and dung density to 22% of baseline levels to achieve a 3.5:1 livestock-to-wildlife ratio led to an approximately 50% reduction in parasite exposures at matrix sites and a much smaller reduction in exposures at water sources, creating a notable jump (1.4–1.6-fold increase) in relative importance of water sources for parasite exposures. Further simulated reductions in wildlife activity and dung density to achieve an 8:1 livestock-to-wildlife ratio led to further increases (1.6–1.8-fold increase) in the relative level of exposure at water (figure 3*b*). However, we also note that simulated wildlife loss led to overall reductions in parasite exposure because host and parasite density decreased.

When we considered drinking water as an additional parasite exposure route, patterns were similar across species, with water becoming a more important hotspot of potential parasite exposure than when considering grazing alone. Specifically, the hotspot effect (ratio of exposures at water versus matrix sites) was 1.5–2 times higher for impala, buffalo, zebra and cattle. For giraffe and elephants – species that tended to spend more time drinking water than grazing – the hotspot effect increased by approximately seven- and threefold, respectively (electronic supplementary material, appendix SI, §3, table S6, figure S5).

## 4. Discussion

Much previous work has demonstrated the significant role of superspreading individuals in influencing transmission dynamics for directly transmitted diseases [45]. However, the superspreading potential of landscape hotspots in environmental transmission is less commonly quantified, often because the location of transmission is challenging to pinpoint [13]. Our findings address this knowledge gap and advance previous work [9] by illustrating the extent to which water sources have the potential to act as environmental hotspots for both within-species and across-species parasite exposures. We show that after accounting for interspecific parasite sharing among ruminants, the small fraction of land surrounding water sources (0.5% in this system) accounts for a significant proportion of potential parasite exposures (3–18% within 50 m, and 9–32% within 150 m, depending on host species), and that this effect is driven by cattle when they outnumber wild ruminants and are not treated with anthelmintics (figure 3*c*).

We also found that accounting for interspecific parasite sharing was most important for species that were closely related to those with high parasite outputs (high faecal egg counts and dung density in the environment) around shared water sources. Specifically, our finding that untreated cattle would have the largest effects on parasite exposures for other species is likely to be applicable in many other contexts where cattle share food and water resources with other ruminants and dominate an ecosystem, a common scenario across rangelands globally [46]. This effect increases with shifts to increasingly cattle-dominated communities. For example, in a scenario in which wildlife biomass at Ol Pejeta Conservancy falls to only an eighth of that of cattle (the 2013 scenario across Kenya [4]), we estimated that the relative level of parasite exposure at water versus matrix areas would nearly double compared with current levels (figure 3*b*).

These findings also highlight the need to consider parasite sharing dynamics at interfaces between wildlife and the several other livestock species, many of which are increasing globally and regionally [1,4] and are known to share a large number of pathogens with wildlife [47]. For example, wild ruminants may also be susceptible to gastrointestinal parasite sharing with sheep and goats, particularly when these livestock are numerous or untreated. While historical challenges in species-level gastrointestinal parasite identification have hindered quantifications of faecal-oral parasite sharing, it is nonetheless clear that interspecific parasite sharing is an issue for these taxa. Notably sheep and goats are thought to spread several directly transmitted parasites including sarcoptic mange [48] and conjunctivitis [49] to sympatric wild ungulates and several wild ungulates can have high prevalence of gastrointestinal nematodes, notably *Haemonchus contortus* [10], that are highly pathogenic to sheep and goats.

While exposures to gastrointestinal parasites have been shown to correspond to resulting infections in a variety of systems, variation in susceptibility determines eventual infections [50,51]. We used several different estimates of parasite sharing as a proxy for susceptibility, including observed parasite infection data from a nearby study system, but experimental tests of the influence of livestock on both exposures and resulting gastrointestinal parasite infections in wildlife will provide practical management information and insights into parasite susceptibility across species, which is notoriously difficult to quantify for wildlife [52]. Our findings suggest that in areas where wildlife and cattle strongly overlap and where cattle are not regularly de-wormed, other ruminants will have a higher relative abundance of parasites known to infect cattle than their counterparts in areas with no cattle interaction. While controlled studies are needed to confirm this pattern, recent research in France found that the gastrointestinal nematode community of wild roe deer was dominated by generalist parasites that were also found in overlapping sheep [53], although there were low levels of parasite sharing among sheep and several cervids in Sweden [54].

Species-specific variation in parasite exposure across a landscape may also provide further insights into observed parasite community composition differences among hosts. For example, at matrix sites, intraspecific exposures for buffalo and impala accounted for at least 25% of total exposures, while giraffes were far more likely to be exposed to parasites from other host species than from conspecifics, a finding that was emphasized by our supplementary analysis that considered parasite risk via both grazing and drinking (electronic supplementary material, appendix SI). If infection probabilities among host species are proportional to exposure probabilities, this may explain why giraffes had no unique (specialist) parasite species in the metabarcoding analysis [10], and it suggests that strongylid parasite composition in giraffes may be heavily influenced by the surrounding herbivore community, especially when giraffes are relatively uncommon. Indeed, previous work has suggested that in general, rare host species tend to harbour fewer specialist parasites compared with abundant hosts, potentially because low population densities of rare species result in very few successful transmissions of specialist parasites [55].

Our conclusions were largely consistent across the different methods used to estimate the degree of parasite sharing, exemplifying the strong and well-documented relationship between phylogeny and gastrointestinal parasite infections (e.g. [30,31]). While metabarcoding data is considerably more time-consuming and expensive to collect and analyse than data obtained from host-parasite databases or phylogenetic inference, we conclude that it will be extremely useful for context-specific sharing estimates. However, when analytical resources are limited, we also found that estimates of parasite sharing based on prior studies corresponded with metabarcoding and phylogeny, suggesting that this method may be reliable in similar applications when a host species is well studied. However, literature-based estimates of parasite sharing may be severely underestimated for relatively rare or understudied species [24], leading to potential underestimates of the effect of the surrounding host community on parasite dynamics.

Despite similarity in patterns of cattle contributions to parasite exposures across the three methods of calculating parasite sharing, one significant difference was the role of zebras as sources of parasite exposure for other animals. When combined with their large parasite output and dung density in the environment, even the very small degree of sharing estimated from the literature and phylogenetic methods led to a considerable number of potential exposures for ruminants, especially giraffes. While metabarcoding data did not indicate that ruminants and equids readily shared parasites, equids are known to be hosts of *Trichostrongylus axei*, a nematode capable of infecting an extremely broad range of hosts, including humans [56], with high prevalence and infection intensity [57]. Therefore, we recommend that site-specific data on parasite infections (e.g. using increasingly common nemabiome methods [58]) be used to estimate potential exposures in a given system, and that this information could be paired with literature and phylogenetic methods to determine risk under additional plausible scenarios.

Lowering cattle parasite density in the environment may be achieved by either reducing cattle density or treating cattle with anthelmintics. Given that cattle accounted for a large share of potential interspecific parasite exposures in our system, it seems likely that anthelmintic application could be a highly effective way to decrease parasite exposures for other animals and alter relative levels of exposure at shared resources (figure 3*b*). Indeed, at Ol Pejeta Conservancy, cattle are regularly treated to reduce parasite infections, and the results of our simulations suggest that this practice has had significant benefits for other wild ruminants in the system. In other areas where helminthic disease is identified as a major threat to wildlife, livestock treatment might thus be an effective conservation intervention. Conversely, our findings also suggest that rising anthelmintic resistance in livestock [30] will also have implications for wild animals that share these parasites. While relatively little is known about the prevalence and virulence of resistant parasites in wild herbivores [59], recent developments in high-throughput molecular screening for genotypes that confer resistance (e.g. [60,61]) will enable estimates of shared anthelmintic-resistant parasites at wildlife livestock interfaces. This is particularly important if wild animals experience altered virulence from resistant parasites or transport resistant parasites among livestock herds via ranging patterns [59,62].

Our findings are also relevant amid recent recognition of the potential for disgust to play a role in modulating parasite exposure [63,64]. Disgust behaviours, such as dung avoidance [65], are thought to be a means for wildlife to reduce parasite exposure. Rapid changes in the relative riskiness of certain habitats (e.g. large increases in livestock density) may lead to a mismatch in evolved avoidance behaviours and actual exposure levels, with animals seeking resources according to intraspecific parasite transmission risk despite high interspecific parasite transmission risk. Alternatively, if animals do indeed recognize and respond to increased habitat riskiness, the energy costs in avoiding needed resources may have substantial impacts on ranging patterns, as explored in recent modelling work [66]. Given the considerable risks that parasite exposure poses for many wild ungulates, it would also be important to test for a signature of interspecies faecal avoidance, and to understand how and if species adjust their avoidance behaviours as parasite exposure levels fluctuate across space and time.

## 5. Limitations

We made several assumptions to enable estimates of parasite exposures across multiple host species, parasite species and landscape contexts. Firstly, we assumed that each similarity value (from metabarcoding, literature and phylogeny) was approximately proportional to the species composition of parasites in dung. Our results were similar across these methods, indicating that they are robust to shifts in the relative abundance of different parasites, but future work linking egg burden, worm burden and relative read abundance from metabarcoding data will allow for more refined estimates, including individual-level variation and variation across seasons.

We also assumed that parasite mortality in the environment was consistent at matrix sites and water sources. However, parasitic nematodes are sensitive to temperature and relative humidity [67,68], and any consistent difference in these conditions between water sources and matrix sites will alter the relative levels of parasite exposure [9]. Differences in microclimates arising from differential vegetation responses to herbivore gatherings at water sources [69], as well as microclimates in dung of different species may also alter parasite mortality and should be considered when assessing relative levels of exposure. Additionally, we chose to measure parasite risk using egg density in dung rather than larval density on grass because it allowed us to identify different host species as sources of generalist parasites. However, incorporating measurements of larval parasite density over different times of the day could provide data on both the relative mortality of parasites in the environment and the risk of transmission at finer time scales.

One aspect of our non-invasive approach is that host individuals could not be tracked over space and time; thus, individual-level estimates of exposure could not be estimated, including at other areas of the landscape where animals may aggregate, such as grazing lawns [70]. While recent developments in animal tracking and analytical methods to assess direct and indirect contacts from GPS data provide high-resolution information on movement and risk [71], analyses are typically restricted to a relatively small number of collared individuals that represent a small fraction of a population; and thus, interspecific contact rates are challenging to measure on a large scale. However, combining the non-invasive multi-species methods described here with individual tracking will undoubtably improve our ability to estimate, monitor and test disease transmission predictions at multiple scales across landscapes.

## 6. Conclusion

Our findings demonstrate that when cattle are abundant and receive relatively little parasite control, they can strongly influence gastrointestinal parasite dynamics for overlapping and related wildlife species, particularly at aggregation sites. This suggests that increases in livestock likely have important consequences for wildlife health, and possibly also wildlife behaviour, if this prompts avoidance behaviour from wildlife around key resources. Critically, this risk is not evenly distributed across wildlife but can be effectively predicted with a variety of parasite sharing network approaches. Identifying this risk spatially and taxonomically will enable efficient management, most notably including anthelmintic treatment of livestock in areas where shared environmentally transmitted parasites pose health risks to wildlife. Such treatments may be especially important in a world where rare or declining wildlife must increasingly share spaces with livestock. Together, these findings demonstrate the significant role that humans may play in influencing parasite exposures for wildlife via livestock and resource management.

## Data Availability

All data and code necessary to replicate the analyses are available from the Dryad Digital Repository: https://doi.org/10.5061/dryad.vdncjsz28 [72]. Supplementary material is available online [73].
